# Routine postoperative admission to the neurocritical intensive care unit after microvascular decompression: necessary or can it be abandoned?

**DOI:** 10.1007/s10143-022-01910-4

**Published:** 2022-12-09

**Authors:** Gökce Hatipoglu Majernik, Filipe Wolff Fernandes, Shadi Al-Afif, Hans E. Heissler, Thomas Palmaers, Oday Atallah, Dirk Scheinichen, Joachim K. Krauss

**Affiliations:** 1 Department of Neurosurgery, Hannover Medical School, Carl-Neuberg-Straße 1, 30625 Hannover, Germany; 2 Department of Anaesthesiology and Intensive Care, Hannover Medical School, Hannover, Germany

**Keywords:** Hemifacial spasm, Intensive care, Microvascular decompression, Trigeminal neuralgia

## Abstract

Postoperative neurocritical intensive care unit (NICU) admission of patients who underwent craniotomy for close observation is common practice. In this study, we performed a comparative analysis to determine if there is a real need for NICU admission after microvascular decompression (MVD) for cranial nerve disorders or whether it may be abandoned. The present study evaluates a consecutive series of 236 MVD surgeries performed for treatment of trigeminal neuralgia (213), hemifacial spasm (17), vagoglossopharyngeal neuralgia (2), paroxysmal vertigo (2), and pulsatile tinnitus (2). All patients were operated by the senior surgeon according to a standard protocol over a period of 12 years. Patients were admitted routinely to NICU during the first phase of the study (phase I), while in the second phase (phase II), only patients with specific indications would go to NICU. While 105 patients (44%) were admitted to NICU postoperatively (phase I), 131 patients (56%) returned to the ward after a short stay in a postanaesthesia care unit (PACU) (phase II). Specific indications for NICU admission in phase I were pneumothorax secondary to central venous catheter insertion (4 patients), AV block during surgery, low blood oxygen levels after extubation, and postoperative dysphagia and dysphonia (1 patient, respectively). There were no significant differences in the distribution of ASA scores or the presence of cardiac and pulmonary comorbidities like congestive heart failure, arterial hypertension, or chronic obstructive pulmonary disease between groups. There were no secondary referrals from PACU to NICU. Our study shows that routine admission of patients after eventless MVD to NICU does not provide additional value. NICU admission can be restricted to patients with specific indications. When MVD surgery is performed in experienced hands according to a standard anaesthesia protocol, clinical observation on a neurosurgical ward is sufficient to monitor the postoperative course. Such a policy results in substantial savings of costs and human resources.

## Introduction

It has been common neurosurgical practice to admit patients after elective craniotomies routinely to the neurocritical care unit (NICU) for monitoring for 12–24 h after surgery [1, 2]. However, the true benefit of this practice has been questioned, especially when facing hospital costs, capacities, and logistics. The value of routine admission to NICU after elective craniotomies has been re-evaluated in different fields of neurosurgery, including mainly neurooncology and functional neurosurgery [3–17].

The necessity of the admission of patients after elective vascular neurosurgery, however, has received less attention. Microvascular decompression (MVD) according to the technique of Jannetta has become a well-defined standard neurosurgical procedure for the treatment of cranial nerve disorders such as trigeminal neuralgia, hemifacial spasm, vagoglossopharyngeal neuralgia, paroxysmal vertigo, and pulsatile tinnitus [18–27]. MVD has a relatively high success rate early postoperatively, and the frequency of complications, in general, is relatively low, while at long-term follow-up, 70 to 80% of patients have a sustained benefit [26, 28–33]. The practice for routine NICU admission after MVD has varied greatly from center to center [6, 30, 34–39].

Currently, there is no consensus regarding the postoperative management and monitoring of patients after MVD. This prompted us to perform a comparative study to investigate the real need and the practical value of routine postoperative admission to NICU after MVD. A before/after approach was used to compare two groups of consecutive patients over two phases—routine NICU admission versus short stay in the postanaesthesia care unit (PACU) to control bias. Our hypothesis was that routine postoperative NICU admission after uneventful MVD may not yield additional clinical benefit.

## Materials and methods

Over a 12-year period, 236 MVD surgeries were performed for treatment of trigeminal neuralgia (213), hemifacial spasm (17), vagoglossopharyngeal neuralgia (2), paroxysmal vertigo (2), and pulsatile tinnitus (2) by the senior author (JKK) at the Department of Neurosurgery at Hannover Medical School. For all patients detailed surgical and anaesthesia, reports were available. During the first phase of the study, patients were routinely admitted to NICU after MVD surgery (phase I), while during the second phase (phase II), patients had a short stay in PACU, and admission to NICU would have been reserved for patients with perioperative complications.

Preoperative American Society of Anaesthesiologists (ASA) physical status grades were assessed in all patients in order to stratify the risk for intra- and postoperative complications according to the following classification: ASA I: normal healthy patient; ASA II: patients with a mild systemic disease; ASA III: patients with severe systemic disease that is not life-threatening; ASA IV: patients with a severe systemic disease that is a constant threat to life; and ASA V: moribund patient who is not expected to survive without the operation [40]. Furthermore, medical comorbidities were assessed, including arterial hypertension, coronary heart disease, cardiac arrhythmia, congestive heart failure, chronic obstructive pulmonary disease (CODP)/asthma, thyroid disorders, gastroesophageal reflux disease, diabetes mellitus, and chronic kidney disease.

Standard departmental assessment and surgical techniques for MVD have been described in detail elsewhere [41–46]. All surgeries were realized under general anaesthesia. Briefly, MVD was performed in a modified “semi-concorde”-prone position. The head was fixed in a Mayfield® head clamp slightly flexed and rotated by 45 degrees to the contralateral side. A retroauricular curvilinear skin incision was made, and a small lateral suboccipital osteoclastic craniotomy was achieved. The dura was exposed medial to the sigmoid sinus just below the transverse sinus. After dural opening, the cerebellum was gently retracted for relief of cerebrospinal fluid. Thereafter, under the microscope the cranial nerve to be decompressed was approached. The affected nerve was examined carefully from its entry/exit zone from the brainstem along its course to the skull base to detect any vascular contacts. Arachnoid membranes were dissected carefully, in no case the cranial nerves were “pinched” or injured. In cases with arterial vascular contact, polytetrafluoroethylene (Teflon®) felt was placed between the offending vessel and the nerve to prevent a recurrent neurovascular contact. Veins were mobilized and were also protected by polytetrafluoroethylene (Teflon®) or they were coagulated if feasible. Fibrin glue was used in the early study period. The dura was closed in a water-tight fashion, and the osseous defect was covered with polymethylmethacrylat.

The anaesthesiological protocol in phases I and II followed a standard operating protocol, respectively. Phase I: total intravenous anaesthesia with propofol, remifentanil, and atracurium followed by endotracheal intubation. At least two peripheral venous catheters, an arterial line, and a central venous line were installed. All patients received a gastric tube and a urinary catheter. After the procedure, patients were admitted to the NICU with overnight stay. Phase II: total intravenous anaesthesia with propofol, remifentanil, sufentanil, and atracurium followed by endotracheal intubation. At least two peripheral venous catheters and an arterial line and only if deemed necessary, a central venous line, were installed. All patients received a gastric tube and a urinary catheter. Patients were admitted to the PACU for a short stay.

Postoperatively, patients were turned to the supine position, and a sonographic examination was achieved to screen for pneumothorax if a central venous line had been inserted. Patients were extubated in the operating room.

After extubation, a postoperative neurological examination was made to screen for side effects. In general, patients were discharged 2 or 3 days after surgery. After discharge from the hospital, patients were scheduled for clinical follow-up examinations 3 months after surgery.

Statistical analysis was performed by JMP® software, Version 16 (SAS Institute Inc., Cary, NC, 1989–2019). *P* values < 0.05 were considered statistically significant. Unless otherwise noted, Fisher’s exact test was used to determine the statistical impacts on results. Alternatively, contingency analysis (chi^2^ test) was conducted on cross-tables (ASA) and Student’s *t*-test on age differences.

## Results

Overall, there were 138 women (58%) and 98 men (42%) with a mean age of 59.1 years (range, 20–87 years; median 61) at surgery. During phase I, all 105 patients (44%) were admitted to NICU postoperatively, whereas in phase II, all 131 patients (56%) returned to the ward after staying in PACU. Patient cohorts in phase I and phase II were comparable. Patients in phase I consisted in 57 women (54%) and 48 men (46%) with a mean age at surgery of 60.1 years (range 30–87; median 61). The distribution of ASA scores was ASA I (12 patients; 11%), ASA II (74 patients; 71%), and ASA III (19 patients; 18%). Patients in phase II consisted in 81 women (62%) and 50 men (38%) with a mean age at surgery of 58.3 years (range, 20–84; median 61). The distribution of ASA scores was ASA I (18 patients; 14%), ASA II (95 patients; 72%), ASA III (17 patients; 13%), and ASA IV (1 patient; 1%).

Seven patients in phase I had specific indications for NICU admission: pneumothorax secondary to central venous catheter insertion (4 patients), AV block during surgery (1 patient), low blood oxygen levels after extubation (1 patient), and postoperative dysphagia and dysphonia (1 patient) after MVD for vagoglossopharyngeal neuralgia. None of the patients in phase II had specific indications to go to NICU, and there was also no secondary referral from PACU to NICU.

In the next step, for further comparison of demographic data and risk factors between groups, the patients who had specific indications for NICU admission in phase I were excluded (*n* = 7). Demographic data of the remaining 98 patients of phase I are shown in Table 1 compared with the data of patients from phase II. In addition, the distribution of ASA scores and the comparative frequency of comorbidities are shown in Table 1. There were no statistically significant differences between groups.

**Table 1:** Comparison of demographic data and comorbidities in patients from phase I (routine admission to NICU) and phase II (short stay in PACU)

	Phase I (*n* = 98)^2^	Phase II (*n* = 131)	*p* value^*^
Age	60.6	58.3	0.21
Gender F/M (%)	51/47 (52%/48%)	81/50 (62%/38%)	0.13
ASA scores^1^			0.50
ASA I	12 (12%)	18 (14%)	
ASA II	68 (69.5%)	95 (72%)	
ASA III	18 (18.5%)	17 (13%)	
ASA IV	0	1 (1%)	
Comorbidities			
Arterial hypertension	36	49	0.91
Coronary heart disease	3	7	0.39
Cardiac arrhythmia	3	9	0.18
Congestive heart failure	5	2	0.11
COPD/asthma	7	15	0.26
Thyroid disorders	7	15	0.26
Gastroesophageal reflux disease	11	14	0.89
Diabetes mellitus	13	5	0.08
Chronic kidney disease	4	1	0.08

*NICU*, neurocritical intensive care unit

*PACU*, postanaesthesia care unit

^1^ASA scores: *ASA I*, a normal healthy patient; *ASA II*, a patient with a mild systemic disease; *ASA III*, a patient with a severe systemic disease that is not life-threatening; *ASA IV*, a patient with a severe systemic disease that is a constant threat to life

^2^Note that 7 patients with specific indications for NICU admission in phase I were excluded from this analysis

^*^p < 0.05 was considered statistically significant

There were no intraoperative surgical complications neither in phase I nor in phase II. No patient underwent a secondary revision surgery within 24 h after surgery. One patient in phase I, a 69-year-old woman who underwent MVD for HFS, had a mild gait ataxia associated with a small hemorrhage at the site of the Teflon® [45]. There were no symptomatic postoperative hemorrhages in phase II.

## Discussion

Routine postoperative admission to the intensive care unit (ICU) after elective craniotomies has been founded on the assumption that patients would benefit from the early detection of postoperative complications including brain edema, hemorrhage, seizures, and cardiac or respiratory failure [8, 13]. While this has been common practice for decades in many countries worldwide, only more recently this concept has been challenged [10, 37, 47]. The scarcity of healthcare resources, particularly ICU beds and trained medical personal, which has been especially accentuated since the COVID19 pandemic [48], demands a need to re-evaluate this practice in various fields of neurosurgery [3, 4, 10].

Although a pioneer study on this topic was already published in the early 1980s, it took decades until the subject received more attention. Knaus and colleagues had shown that patients admitted to the ICU after uncomplicated elective neurosurgery rarely required active interventions [15]. The majority of patients actually received only “intensive monitoring.” The authors raised the question whether such monitoring would require admission to the ICU. These lines of thought were followed in a later study by Zimmermann et al. [16]. Using data from the APACHE III study of 17,440 ICU patients, the authors identified 3000 patients admitted to ICU for neurological care; however, 95.8% of patients received only “intensive monitoring” services.

Subsequently, few studies investigating the need for ICU admission after elective craniotomy became available, concentrating mainly on neurooncology. Beauregard et al. showed that patients who were admitted to ICU postoperatively did not have fewer complications compared to those transferred directly to the ward (3). In another study, Mirza et al. indicated that in a series of 397 patients who underwent resection of intraaxial brain tumors, the majority of patients were not routinely admitted to ICU postoperatively, and only 4 patients (1.1%) developed secondary complications, requiring transfer to ICU from floor (9). Both diabetes and older age were thought to indicate a need for postoperative admission to ICU after elective craniotomy (6).

Little information is available on the true necessity and on current practice of NICU admission of patients undergoing MVD. Practices seem to vary highly not only from country to country, but also within communities. While in many publications on the postoperative care of MVD patients no information whether ICU admission was standard of care or not was provided, it appears that many institutions still follow the concept that ICU overnight after MVD would provide additional safety [30, 34–36, 38].

In a study on 44 patients who underwent MVD mainly for TN published in 2014 by McLaughlin et al., the authors showed that one of the main factors contributing to the global cost of hospital stay was postoperative admission to ICU [36]. While patients were admitted routinely to ICU initially, the authors suggested to “revise” clinical routines, particularly regarding postoperative admission to ICU after MVD. They indicated that more studies would be needed to identify when targeted bed assignment to ICU is required reserving beds for either intensive care or intensive monitoring. In a recent study comparing two institutions with different practices for NICU admission after MVD (190 patients without NICU stay versus 90 patients with routine admission), it was shown that postoperative complications requiring ICU stay were comparable between groups (7 versus 5 patients) and that coronary artery disease was a predictor in both institutions [38]. The authors concluded, however, that their results should be interpreted carefully with regard to institutional predictors and that the results may be limited to centers with more experience and higher or moderate loads of MVD surgery.

As outlined above, ICU bed assignment and length of stay are among the most expensive factors in surgical care in MVD [36]. Strategies aiming to bypass postoperative ICU can save considerable costs, which may vary considerably across hospitals worldwide. At Hannover Medical School, postoperative NICU stay is equivalent to €2200 per day of care. Young and colleagues, using a safe transitional pathway for elective craniotomies, including brain tumor resections, MVD, and Chiari malformation decompressions, showed savings of $4000 per case [47]. Also, Beauregard demonstrated in 429 elective craniotomies significant average total cost savings of $4026 for floor versus ICU patients, including direct and indirect cost components (3). Additionally, a study from the Netherlands showed that a policy of “no ICU, unless” in elective supratentorial craniotomies could result in savings of €1953 in mean total costs per case [49].

We conducted the study in a before/after approach, which has advantages as compared to a study with a historical cohort [50]. On the other hand, the Hawthorne effect has to be considered in such a study design [51], which may have been relevant in the reduction of postoperative complications such as pneumothorax.

Our study demonstrates that a radical change of paradigm regarding NICU admission after MVD surgery is possible given certain premises are followed. We are cognizant that the results of our study may not be generalized, since it has been performed under particular circumstances with all surgeries having been done by a dedicated and experienced senior surgeon. Most importantly, however, the close collaboration between the neuroanaesthesiological and the neurosurgical team according to standard operation protocols with enhanced high-value medical care is indispensable.

## Conclusion

In the present study, we show that routine admission to NICU for patients who undergo MVD for cranial nerve disorders does not yield additional value. Postoperative monitoring for a short period can be performed in PACU and patients may be referred to the ward early.

## Data Availability

Not applicable.
